# Inorganic Phosphate Modulates the Expression of the NaPi-2a Transporter in the *trans*-Golgi Network and the Interaction with PIST in the Proximal Tubule

**DOI:** 10.1155/2013/513932

**Published:** 2013-02-14

**Authors:** Miguel A. Lanaspa, Yupanqui A. Caldas, Sophia Y. Breusegem, Ana Andrés-Hernando, Christina Cicerchi, Moshe Levi, Victor Sorribas

**Affiliations:** ^1^Department of Medicine, University of Colorado, and VA Eastern Colorado Health Care System, Denver, CO 80220, USA; ^2^Department of Toxicology, University of Zaragoza, 50013 Zaragoza, Spain

## Abstract

Inorganic phosphate (Pi) homeostasis is maintained by the tight regulation of renal Pi excretion versus reabsorption rates that are in turn modulated by adjusting the number of Pi transporters (mainly NaPi-2a) in the proximal tubules. In response to some hormones and a high dietary Pi content, NaPi-2a is endocytosed and degraded in the lysosomes; however, we show here that some NaPi-2a molecules are targeted to the *trans*-Golgi network (TGN) during the endocytosis. In the TGN, NaPi-2a interacts with PIST (PDZ-domain protein interacting specifically with TC10), a TGN-resident PDZ-domain-containing protein. The extension of the interaction is proportional to the expression of NaPi-2a in the TGN, and, consistent with that, it is increased with a high Pi diet. When overexpressed in opossum kidney (OK) cells, PIST retains NaPi-2a in the TGN and inhibits Na-dependent Pi transport. Overexpression of PIST also prevents the adaptation of OK cells to a low Pi culture medium. Our data supports the view that NaPi-2a is subjected to retrograde trafficking from the plasma membrane to the TGN using one of the machineries involved in endosomal transport and explains the reported expression of NaPi-2a in the TGN.

## 1. Introduction

The proximal tubule of the kidney is the main anatomical structure that maintains inorganic phosphate (Pi) homeostasis, by virtue of its ability to fine-tune the Pi reabsorption rate. Renal Pi excretion and reabsorption depend on the abundance of specific Na-dependent Pi transporters expressed in the brush-border membrane of the proximal tubular epithelial cells [1]. This abundance is controlled by several hormonal and nonhormonal agents, including phosphatonins, parathyroid hormone (PTH), and dietary Pi concentration [2]. Two type II Pi transporters (NaPi-2a and NaPi-2c) and two type III transporters (PiT-1 and PiT-2) are expressed in the proximal tubule of the nephron. Of them, NaPi-2a is responsible for more than 90% of Pi reabsorption under standard conditions, as estimated using physiological approaches [3]. NaPi-2a has also been the most characterized Pi transporter, and the cellular mechanisms that regulate the apical expression of NaPi-2a in response to PTH or dietary Pi concentration have been extensively studied. 

Acute changes in reabsorption rate are achieved within minutes by the fast insertion into, or retrieval of, Pi transporters from the membrane, mainly NaPi-2a. Potential post-translational modifications, such as phosphorylation that could explain this regulatory mechanism, have not been described in the case of NaPi-2a. For example, upon binding of PTH to corresponding PTH receptors of proximal tubular cells, most NaPi-2a transporters are endocytosed through clathrin-coated pits and early endosomes and degraded in lysosomes [4, 5]. This internalization is dependent on the rearrangement of microtubules into dense bundles of apical-to-basal orientation [6]. Microtubules are also involved in another acute regulation mechanism, that is, the adaptation to acute changes in dietary Pi concentration, but this mechanism has been less studied than the effect of PTH on the renal Pi transporters [7]. In rodents the expression of NaPi-2a in the brush border membrane of the proximal tubular cells increases within a few hours in response to low dietary Pi diet and decreases after eating high Pi-containing diets [8]. Changes in Pi transport activity and protein abundance of NaPi-2a during adaptation are not very extreme, but they are limited to 100% changes approximately, between minimal and maximal levels of both transport activity and protein abundance [9]. Acute upregulation in response to low Pi diet is independent of transcriptional and translational activities, suggesting the existence of an NaPi-2a storage mechanism that still has to be determined [7]. Similarly to the response to PTH, a high Pi diet induces an acute downregulation of Pi reabsorption which is mediated by the fast internalization of NaPi-2a transporters from the brush border toward early and late endosomes and, again, lysosomal degradation [10]. In all these cases, the translocation of NaPi-2a transporters seems to be dependent on the interaction of NaPi-2a carboxyl-end with PDZ domain-containing proteins [11, 12]. 

The existence of intracellular storage of NaPi-2a has not deserved much attention. Immunofluorescence microscopy analyses have revealed clear colocalization of NaPi-2a with lysosomal and Golgi markers and additional subapical accumulations [7]. The relative expression in the different intracellular compartments varied with the Pi content of the diet, with maximal changes in expression corresponding to the lysosomal compartment after an acute (2–4 hours) adaptation from low-to-high Pi diet. More recently, however, we have described the changes in the expression in the *trans*-Golgi network (TGN) in parallel to the changes in dietary Pi [13].

In this work we have studied the *in vivo* expression of NaPi-2a in the TGN. Expression in TGN changes not only with dietary Pi adaptation, but also by overexpression of a TGN-located PDZ-domain-containing protein, the PDZ-domain protein interacting specifically with TC10, PIST^CAL,  GOPC,  FIG^ [14]. Our findings suggest that NaPi-2a can be subjected to retrograde transport from the apical membrane toward the TGN, a novel mechanism that has been described for different membrane proteins, as an intermediate checkpoint step in the pathway to the degradation in the lysosomes [15]. 

## 2. Materials and Methods

### 2.1. Animal

Acute and chronic adaptations to different Pi diets have been detailed in numerous previous manuscripts (e.g., [8, 16]). Briefly, rats were fed chronically (three days) fodder containing 0.1% (low), 0.6% (control), or 1.2% (high) Pi diets (Provimi Kliba SA, Penthalaz, Switzerland). Experiments involving animals were performed by the authors in Spain, according to European Animal Welfare regulations, and were explicitly approved by the ethical committee of the University of Zaragoza. 

For acute experiments, male Wistar rats (6–8 week old; Janvier SAS, St. Berthevin, France) were supplied food from 08:00 to 10:00 AM each day, after which they had an access to tap water only. They were first conditioned on the high Pi or low Pi diets for 3 days, then switched to the acute dietary regime at 08:00 am (i.e., acute low-to-high Pi, or acute high-to-low Pi), and subsequently sacrificed in triplicates four hours after switching to the acute diet. 

### 2.2. Cell Culture and Transfections

Wild-type Opossum Kidney (OK) cells were grown to confluence in DMEM/Ham'sF-12 (Invitrogen, Carlsbad, CA, USA), 10% FBS, as described in [13]. Transfections and cotransfections were achieved with Lipofectamine 2000 (Invitrogen) when the cells were 90% confluent, following the manufacturer's instructions.

### 2.3. Transport Assays

Radiotracer uptake assays were performed using ^32^P-H_3_PO_4_ as radiotracer, as reported previously [13, 17]. Cells were grown on plastic plates, and uptake was measured in the presence and absence of Na^+^; in this case, Na^+^ was substituted equimolarly by choline. 

### 2.4. Immunoblotting and Coimmunoprecipitation Assays

Western blots were performed as described (e.g., [16]). Proteins were transferred into PVDF membranes and detected using either a polyclonal antibody against NaPi-2a from rat [16], or an anti-PIST (ProSci Inc., Poway, CA, USA) polyclonal antibody.

Coimmunoprecipitation was performed as reported in [18], using the same anti-PIST antibody as for immunodetection and a ProFound Mammalian Coimmunoprecipitation kit (Pierce, Rockford, IL, USA). Brush-border membranes (BBMs) from renal proximal tubular cells were prepared by the Mg^2+^ differential precipitation procedure [19]. For samples containing non-BBM proteins, the pellet obtained by Mg^2+^-precipitation after the first centrifugation was used.

### 2.5. Immunofluorescence Analysis

Rats were fixed *in vivo* by retrograde perfusion, as described in [7, 13]. The *trans*-Golgi network was detected using a monoclonal anti-*β*-COP antibody (Sigma), *β*-actin using a goat anti-*β*-actin antibody (Santa Cruz Biotechnology, Santa Cruz, CA, USA), and tubulin using a monoclonal anti-*α*-tubulin antibody (Sigma). For fluorescence microscopic imaging in OK cells, the open reading frame of rat NaPi-2a and rat PIST was cloned in frame, by high fidelity PCR cloning, into the fluorescent protein-encoding plasmids pEGFP-C1 and pECFP-C1, respectively, (both from Clontech, Mountain View, CA, USA). Immunofluorescent preparations of either tissue sections or OK cells were analyzed with a laser-scanning confocal microscope LSM510 (Carl Zeiss, Thornwood, NY, USA).

### 2.6. Statistical Analysis

Data were compared with Student's *t*-test for two experimental groups. Results were expressed as Mean ± SD. All statistical analysis was performed with GraphPad Prism 5.0 statistical software.

## 3. Results

### 3.1. Expression of NaPi-2a in *trans*-Golgi Network

**Figure 1:**
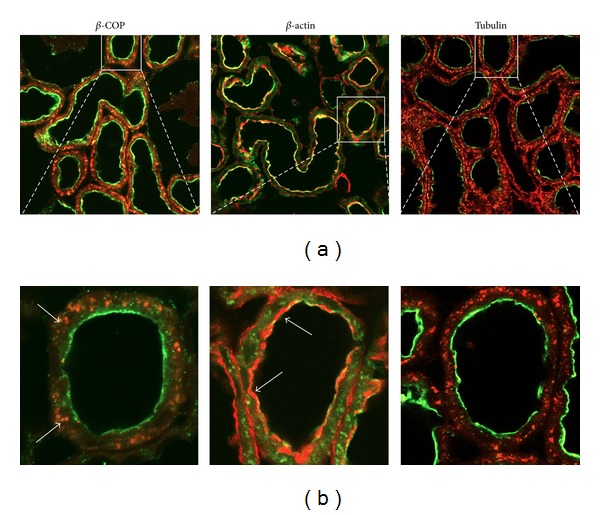
Confocal immunodetection in rat kidney cortex of NaPi-2a (green, Alexa488), *β*-COP (red, Alexa568), *β*-actin (red, phalloidin-rhodamine), and tubulin (red, Alexa 568). NaPi-2a colocalizes with *β*-COP intracellularly, and with *β*-actin in the BBM (see arrows, orange merge). NaPi-2a does not colocalize with tubulin (only green and red are visible, under steady-state conditions). (a) Low magnification of kidney cortex; (b) high magnification to show single proximal tubules.

In addition to the main expression of NaPi-2a in the brush-border membrane of proximal tubular cells, this Pi transporter also exhibits a perinuclear staining, which is compatible with a specific expression in the *trans*-Golgi network [7, 13]. Consequently, we first checked whether the intracellular expression of NaPi-2a could be colocalized with *β*-COP, a classical marker of the *trans*-Golgi. In animals fed a control Pi diet (0.6% Pi), immunofluorescence of rat kidney sections showed that, as expected, NaPi-2a was mostly expressed in the brush-border membrane of the proximal tubular epithelial cells, but intracellular staining could also be observed (Figure 1). The intracellular expression colocalized completely with *β*-COP. Thus, under this dietary control condition the intracellular expression of NaPi-2a could now be defined as the *trans*-Golgi network.

### 3.2. Colocalization of NaPi-2a with PIST in the *trans*-Golgi Network

**Figure 2:**
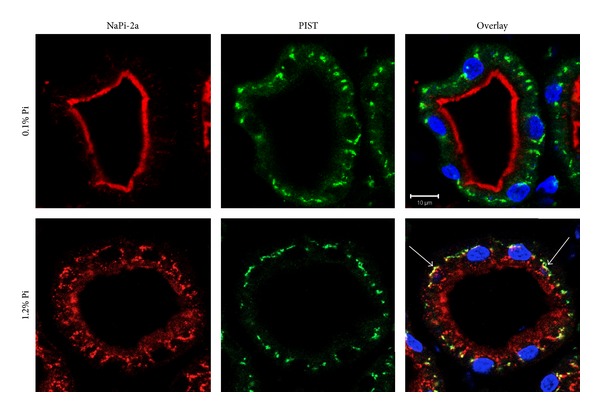
Expression of NaPi-2a (red) and PIST (green) in rat proximal tubules fed chronically (24 h) a low (0.1%) or high (1.2%) Pi diet. Colocalization only takes place intracellularly in high Pi diet fed animals (arrows). Nuclei stained with DAPI (blue).

NaPi-2a expression and subcellular localization are dependent on the interaction of the NaPi-2a carboxyl end with PDZ-domain-containing proteins, such as NHERF1 and PDZK1. PIST is a PDZ protein that localizes mostly to the *trans*-Golgi network, so we sought to determine whether interaction with PIST could explain the expression of NaPi-2a in the TGN. To facilitate the expression analysis the study was performed under two dietary conditions known to modify the expression of NaPi-2a in the apical membrane: 24 hours adaptation to low (0.1%) and high (1.2%) Pi-containing diets. As expected, under Pi deprivation conditions (0.1% Pi diet) practically all signal of the immunofluorescent-labeled NaPi-2a was visualized in the brush-border membrane, and no colocalization with the PIST protein could be observed either in the brush border or in intracellular localizations (Figure 2). However, when the animals were chronically fed a high Pi content diet (1.2% Pi), NaPi-2a was very weakly expressed in the apical membrane, but the intracellular staining of this Pi transporter was increased, showing a clear, but partial colocalization with PIST (Figure 2; see arrows). 

### 3.3. Interaction of NaPi-2a and PIST

**Figure 3:**
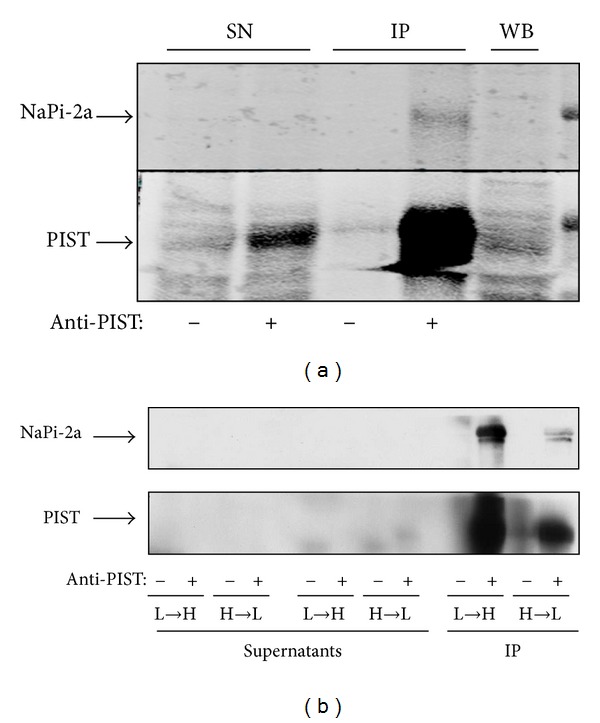
Coimmunoprecipitation assays. (a) Coimmunoprecipitation of PIST and NaPi2a using rat kidney homogenates from rats fed a control diet, and with an anti-PIST antibody. (b) Coimmunoprecipitation in rats acutely adapted for 4 hours from low to high (L-H) and from high to low (H-L) Pi diet, using non-BBM homogenates. SN: supernatant (nonprecipitated samples); IP: immunoprecipitated; WB: Western blotting.

Based on the colocalization of NaPi-2a and PIST observed in rat kidney by IF, and on the fact that several interactions between membrane proteins and the PDZ domain of PIST have been described during the last decade [20–23], we next checked whether NaPi-2a was also able to interact with this PDZ protein. Coimmunoprecipitation assays were performed using a polyclonal anti-PIST antibody and rat kidney samples. Initially, coimmunoprecipitation was performed in the total homogenate of kidney cortex from control diet (0.6% Pi) fed rats (Figure 3(a)). Under these conditions, a weak band specific to NaPi-2a was observed, therefore suggesting an interaction between the transporter and PIST. Interaction was then determined under acute adaptations (4 hours only) to the two Pi diets, in order to study not only the steady state conditions established during the chronic adaptations, but also the movements from and to the cell membrane.  Figure 3(b) shows the results of the two conditions that were studied: from chronic low Pi to acute high Pi diets (low-to-high, 0.1% to 1.2%) and from chronic high Pi to acute low Pi diets (high-to-low, 1.2% to 0.1%), following the standard procedure explained in Section 2 (e.g., [9]). After the four hours of acute adaptation the interaction was assayed, revealing that the immunoprecipitated NaPi-2a was more abundant in the low-to-high Pi diet adaptation than in the high-to-low Pi diet; that is, the interaction was favored when NaPi-2a protein is internalized from the brush-border membrane as an adaptation to an increased Pi intake (Figure 3(b)). It is important to clarify that while several proteins are described to interact with the PDZ domain of PIST through their C-termini (see above), we have not demonstrated that this is the case for NaPi-2a, and, therefore, other molecular interactions can occur. 

**Figure 4:**
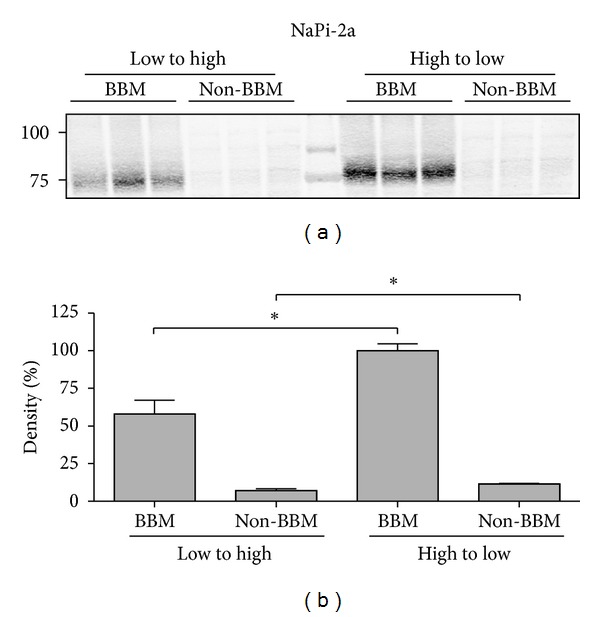
Expression of NaPi-2a using immunoblots of BBM and non-BBM. Bottom chart, corresponding densitometries of the immunosignals with statistical differences.

A Western blot was also performed to determine the abundance of NaPi-2a in the brush-border membrane (BBM) and the non-BBM fractions (Figure 4). Western blot confirmed that NaPi-2a was mainly expressed in the brush border membranes of animals adapted from high-to-low Pi diet, and with minor expression in non-BBM membranes. The low expression is mainly based on the absence of trans-Golgi membrane purifications, because the combination of all nonbrush-border membranes was used as samples.

### 3.4. Effect of PIST Overexpression on NaPi-2a Expression in OK Cells. 

**Figure 5:**
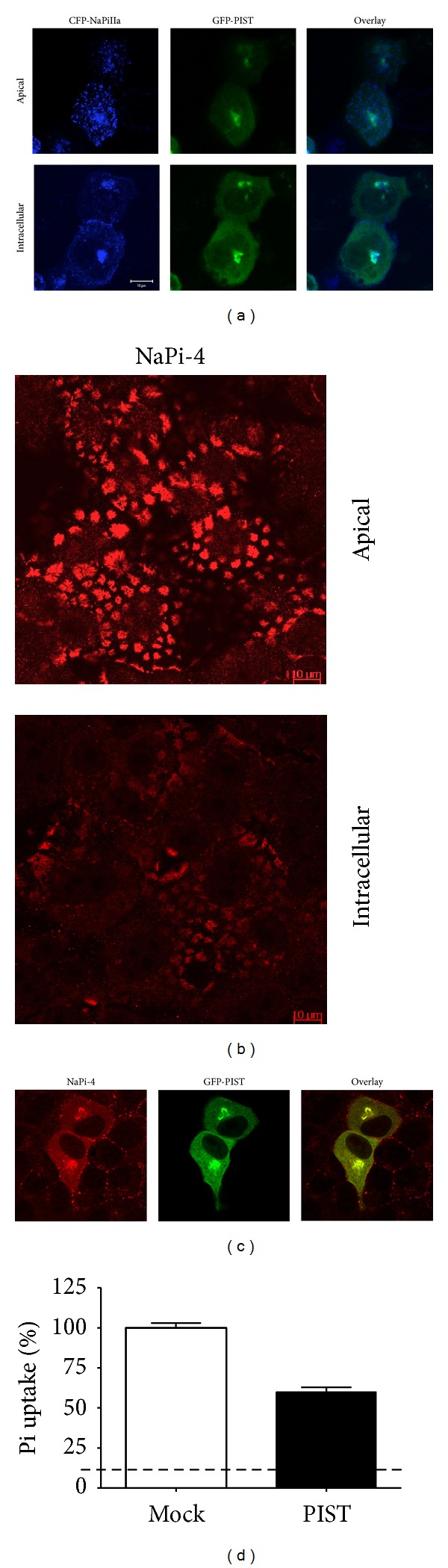
(a) Coexpression of CFP-NaPi2a and GFP-PIST in OK cells. The two proteins colocalize intracellularly at the site of PIST expression. (b) Expression of the endogenous NaPi-2a in OK cells (NaPi-4) is only clearly seen in BBM under control conditions. (c) Overexpression of PIST restricts the expression of NaPi4 to the TGN. (d) Pi transport in OK cells transfected with GFP-PIST is reduced proportionally to the efficiency of PIST transfection. Dashed line: level of Na-independent uptake.

The effect of PIST overexpression was evaluated in Opossum Kidney (OK) cells, a classical *in vitro* model of proximal tubular cells. Transfection of a CFP-NaPi-2a fusion protein plasmid in OK cells reveals that the protein is mainly expressed in the brush-border membrane of the cells, with some minor expression intracellularly, which is compatible with a *trans-*Golgi network localization. When CFP-NaPi-2a is cotransfected with a GFP-PIST plasmid, both fluorescent fusion proteins colocalize intracellularly, again in a place close to the nucleus and compatible with TGN, which in turn, is the normal expression location of PIST (Figure 5(a)).

OK cells express an endogenous NaPi-2a transporter, NaPi-4 [24], which is usually expressed in the brush-border membrane, and intracellular expression is minimal under steady-state conditions (Figure 5(b)). When OK cells were single-transfected with GFP-PIST, resulting in the overexpression of this PDZ protein, NaPi-4 disappeared from the membrane and colocalized with GFP-PIST (Figure 5(c)). Consequently, transfection of PIST also inhibited Na-dependent Pi transport by 40% approximately compared to mock transfected cells, a percentage that is similar to the transfection efficiency of the plasmid (Figure 5(d)).

**Figure 6:**
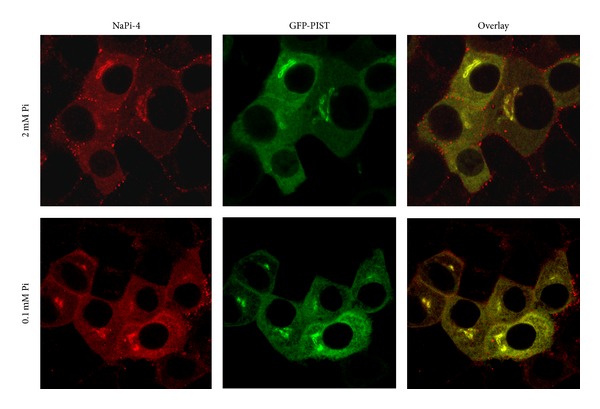
Overexpression of GFP-PIST in OK cells maintains NaPi4 in the trans-Golgi, independently of the concentration of Pi in the cell culture medium (0.1 mM versus 2 mM Pi).

OK cells are an established model for Pi studies that have the ability to adapt to the concentration of Pi in the culture medium similarly to the proximal tubular cells. In the presence of low Pi concentration, the expression of NaPi-4 in the apical membrane increases rapidly [9, 25] as in the mammalian kidney (Figure 2). However, when GFP-PIST-transfected OK cells were adapted acutely (for two hours) by incubating them to either low (0.1 mM) or to high (2 mM) Pi culture medium starting from the control condition, 1 mM Pi, the classical adaptation was blunted by the PIST overexpression. In other words, in PIST-transfected OK cells, NaPi-4 was maintained in the TGN, independently of the concentration of Pi in the culture medium (Figure 6), without the adaptation response of the cells.

## 4. Discussion

Pi reabsorption rate in the renal proximal tubule is the main target mechanism for all hormonal and nonhormonal signals that control Pi homeostasis. Reabsorption rate is determined by the abundance of Pi transporters (mainly NaPi-2a) in the brush-border membrane. Consequently, because Pi transporters seem to be always active and functional (i.e., they are not phosphorylated), changes in Pi transport rate are only the consequence of insertion of transporters into, or internalization from the brush-border membrane. Internalization takes place through endocytosis via clathrin-coated pits and early endosomes, as a response to PTH, the phosphatonins (e.g., FGF23), or adaptation to high Pi diet [4, 5, 7]. The final outcome of the endocytosed transporters is degradation into lysosomes, but this process has not been studied in great detail. The present work is the starting point in a project aimed to study the precise outcome of this Pi transporter after endocytosis-mediated internalization and the role that the PDZ-domain protein PIST might play in this process. 

NaPi-2a interacts through its carboxyl end with several PDZ-domain-containing proteins, such as NHERF-1 and PDZK1 (NHERF3), but the role of these interactions in the subcellular expression and localization in the cell is also not completely understood. NHERF-1 facilitates the residence of NaPi-2a in the brush-border membrane [26], and it is necessary for the adaptation to a low Pi diet [12]. The role of PDZK1 is less evident, but the corresponding RNA is increased during chronic dietary restriction of Pi [24]. The specific role that differentiates PDZK1 from NHERF-1 interaction with NaPi-2a is not clear, beyond a mayor scaffold role beneath the apical membrane [18].

Here we are describing a new interaction between NaPi-2a and a *trans*-Golgi resident PDZ protein, PIST [14]. We have described this interaction using an *in vivo* approach, to avoid the misinterpretations that overexpression (*in vitro*) frequently causes. However, *in vivo *studies have many limitations in understanding the cellular handling of the transporters, and, therefore, parallel *in vitro* cell culture studies are also necessary. With these studies we have shown that the interaction with PIST explains the expression of NaPi-2a in the TGN [7, 13], but also that both the extension of the interaction and the expression of NaPi-2a in TGN, are modified by the Pi content of the diet (Figures 2 and 3). We are not showing direct evidence of the interaction between NaPi-2a and PIST through a PDZ domain interaction. Nevertheless, this is most likely the case, as NaPi-2a almost exclusively interacts with other proteins through its PDZ-binding domain, and the PDZ interacting proteins are the same as other membrane proteins with similar carboxy ends (see below, the CFTR case).

The expression of NaPi-2a in the TGN and the interaction with PIST that we are describing complement the knowledge about the intracellular trafficking of the transporter in the proximal tubular cell. The role of the TGN in Pi homeostasis is of major interest because the TGN is one of the main protein-trafficking checkpoints of the cell at the crossroads between exocytic and endocytic pathways. The TGN, located at the exit face of the Golgi apparatus, sorts cargo proteins (and lipids) into carrier systems towards the plasma membrane, endosomes, and so forth, but it also receives proteins from various endosomal locations, by a process known as retrograde transport [15, 27]. Retrograde transport can occur from the early endosome, the recycling endosome, or from late endosome, and the choice of one or more routes depends on the specific cargo. The different cargos require specific machineries, including the newly described retromer [28]. The TGN decides whether the endocytosed protein is going to either be degraded through the ubiquitin proteasome or through the lysosome pathways, or recycled to the plasma membrane using the recycling endosome. In the case of NaPi-2a, it is not degraded by the proteasome but by the lysosome [7, 10]. However, the existence of a low recycling rate back to the plasma membrane has not been discarded, and therefore the traffic through the TGN should be analyzed in detail.

It seems that for the transition through the TGN the interaction with PIST is a mandatory step, because there is a correlation between the abundance of NaPi-2a expression in the TGN (Figure 2; [14]) and the intensity of the NaPi-2a/PIST interaction (Figure 3). In addition, overexpression of PIST in OK cells induces the internalization of NaPi-2a to the TGN and therefore inhibits Pi transport (Figure 5), but, most important, overexpression also impedes the cells to adapt acutely to a low Pi culture medium (Figure 6). This suggests that the insertion of NaPi-2a transporters from intracellular storage (i.e., adaptation to low Pi concentration) also involves a sorting through the TGN or, alternatively, that overexpression of PIST is impairing the correct functioning of the cargo machinery involved in the movement of NaPi-2a transporters to the plasma membrane. The interaction of PIST seems to be specific with NaPi-2a among Pi transporters, because NaPi-2c does not interact with PIST, and NaPi-2c is not expressed in the TGN [29]. With respect to type III Pi transporters (PiT-1 and PiT-2), they do not contain PDZ-binding domains at their carboxy ends, and the intracellular expression is located in the endoplasmic reticulum [30]. Also, the acute adaptation of NaPi-2a is faster and very different than that of NaPi-2c and PiT-2 [16, 31], a phenomenon that can also be explained by the existence of NaPi-2a recycling. 

The step through the TGN and the interaction with PIST has also been described for different membrane proteins, with different outcome [19–22]. For example, the cystic fibrosis transmembrane conductance regulator (CFTR) interacts with the same PDZ-domain proteins as NaPi-2a (NHERF-1, NHERF-2, PDZK1, and PIST; [19]). When endocytosed, CFTR can be recycled back to the plasma membrane or trafficked to the lysosome, and this depends, at least in part, on the interaction of the carboxyl end of CFTR with the PDZ proteins. In contrast to NHERF-1, PIST reduces cell surface expression of CFTR, retains it in the cell, and promotes degradation in the lysosome [32]. PIST seems to be a component of the trafficking machinery that connects the plasma membrane and the TGN. For this purpose, PIST also needs to interact with a variety of proteins such as TC10 (a Rho family small GTPase) or syntaxin 6 (a member of the Q-SNARE family; [33]). We do not know whether NaPi-2a behaves similarly to CFTR, but it is tempting to suggest that not all NaPi-2a is degraded into the lysosomes as a response to PTH or high Pi concentration, as it would imply a high energetic cost to the cell. Whether NaPi-2a is degraded or recycled, we do not know clearly yet, but in any of both outcomes the TGN will be, most likely, the subcellular structure that will dictate the trafficking route of the internalized transporters.
